# User experience and acceptance of a device assisting persons with dementia in daily life: a multicenter field study

**DOI:** 10.1007/s40520-021-02013-8

**Published:** 2021-11-11

**Authors:** Theresa König, Martina Pigliautile, Oscar Águila, Jon Arambarri, Christophoros Christophorou, Matteo Colombo, Argyris Constantinides, Rosario Curia, Kathrina Dankl, Sten Hanke, Christopher Clemens Mayer, Stefan Moritsch, Markus Müllner-Rieder, Fritz Pernkopf, Christian Schüler, Maria Stillo, Patrizia Mecocci, Elisabeth Stögmann

**Affiliations:** 1 Department of Neurology, Medical University of Vienna, Währinger Gürtel 18-20, 1090 Vienna, Austria; 2 Department of Medicine and Surgery, Section of Gerontology and Geriatrics, University of Perugia, Perugia, Italy; 3 Bidaideak - Sociedad Vasca de Minusválidos, Bilbao, Spain; 4 VirtualWare 2007 S.A., Basauri, Spain; 5 Citard Services Ltd., Nicosia, Cyprus; 6 Innovation Lab, Integris S.P.A., Rende and Pisa, Italy; 7 Design School Kolding, Kolding, Denmark; 8 University of Applied Sciences-FH Joanneum GmbH, Graz, Austria; 9 Center for Health and Bioresources, Biomedical Systems, AIT Austrian Institute of Technology, Vienna, Austria; 10 Bkm Design Working Group, Vienna, Austria; 11 Fritz Pernkopf Industrial Design, Vienna, Austria; 12 Wetouch E.U., Vienna, Austria

**Keywords:** Dementia, Cognitive impairment, Assistive technologies, Digital device, Usability testing, Independent living

## Abstract

**Background:**

Assistive technologies have the potential to facilitate everyday life of people with dementia and their families. Close collaboration with affected people and interdisciplinary research are essential to understand and address the needs of prospective users. In this study, we present the results of the evaluation of such an assistive system prototype.

**Aims:**

Challenges from the patient and caregiver side, technical and design problems and acceptance and usability with regard to our special target group were evaluated.

**Methods:**

MEMENTO, a system of two e-ink tablets and a smartwatch, was tested in the domestic environment of dementia patients. Thirty participants from Italy, Spain and Austria took part in a 3-month field trial and compared the MEMENTO system to traditional strategies in everyday life. Quantitative and qualitative data were collected and frequency of use of the system was monitored.

**Results:**

There were no significant changes in quantitative measurements, such as activities of daily living and caregiver burden over the duration of the 3-month field trial. More frequent usage was significantly correlated with positive attitude towards technology (*r *= 0.723, *p *< 0.05), but not with age. The design of the system was positively emphasized, reducing fear of the technology on the one hand and stigmatization on the other.

**Conclusion:**

We show that a positive attitude towards technology is the essential variable for successful implementation of such systems, regardless of age. Participants showed great interest in digital solutions and agreed that technological systems will help in maintaining independency of persons with cognitive dysfunction in the future.

**Supplementary Information:**

The online version contains supplementary material available at 10.1007/s40520-021-02013-8.

## Introduction

As the world population is aging, the number of individuals with dementia is steadily increasing and by 2050, up to 150 million people are expected to be living with dementia [1], leading to considerable impacts on society and economy. Dementia describes a group of symptoms caused by several different diseases such as Alzheimer’s disease (AD), the most common cause of neurodegeneration in older adults. Clinically, it is characterized by a slowly progressive cognitive decline that mainly affects episodic memory function. In the early stages of dementia, patients are often largely independent but need assistance in certain areas, such as grocery shopping and scheduling appointments. Eventually, in the course of disease, major difficulties in activities of daily living occur. Assistive technologies offer great opportunities to facilitate the everyday life of people with dementia and their caregivers, although there is a lack of high-quality evidence of the effectiveness of such technological aids [2, 3]. Nevertheless, they are expected to contribute in a cost-effective way to prolonged independency of people with dementia and reduced caregiver burden.

Successful implementation of new technology largely depends on user acceptance [4]. Therefore, several theoretical models to measure user acceptance has been established, including the widely used unified theory of acceptance and use of technology (UTAUT) model [5]. Four constructs were identified as determining factors, which are performance expectancy, effort expectancy, social influence and facilitating conditions. The influence of these factors on acceptance was found to be moderated by gender, age, experience and voluntariness of use.

However, many devices are developed without a user-centered design process [6]. The lack of holistic approaches to understand the needs and characteristics of the target group might lead to a reduced acceptance of the technology and consequently the abandonment of the device in the long run [7]. Thus, involvement of the patients in early development processes is essential for identifying their requirements, which is especially important when considering the specific needs of dementia patients [8–10]. In addition, it is necessary to look at these concerns from different perspectives, including medical and design as well as developmental and economic view [11]. Outcomes of such user-centric studies serve as valuable basis for requirement surveys in the development of assistive technologies.

The cross-disciplinary consortia of the MEMENTO project—comprising clinicians, medical and design researchers, hardware and software developers and integration specialists, as well as business experts—developed such an assistive technology in the course of a 3-year international study in Austria, Italy, Spain and Cyprus. Consisting of a self-contained unit of digital notebook and smartwatch, MEMENTO aims to provide a compelling system to support the memory and daily life management of people in early stages of dementia by addressing challenges such as taking medications correctly, organizing, preparing for and keeping appointments, running errands and feeling disoriented. To develop a functional and user-friendly solution with high user acceptance, we designed the system together with dementia patients and their caregivers. A detailed description of the multidimensional design research has been previously published [11, 12]. A report of the first evaluation of the MEMENTO prototype in the context of lab trials at the three clinical sites in Austria, Italy and Spain has been submitted for publication (Pigliautile et al. manuscript in revision). In contrast to the lab trials, the field trials described in the present study took place in the participants’ homes, as the evaluation in terms of user experience and acceptance for such systems in a familiar environment is of uttermost importance. Thus, the main aim of the current study was to assess how well the device is perceived by the participants in everyday life and which factors play a role for acceptance and rejection. In addition, the status of the patients at the beginning of the study was evaluated and possible changes were monitored during the study. Thereby, we aim to provide a reference for current and future studies on the development of assistive technologies for people with dementia.

## Materials and methods

### Study participants

Participants were recruited at three international centers: at the dementia outpatient clinic of the Department of Neurology of the Medical University of Vienna (MUV) in Austria, the Department of Medicine and Surgery, Section of Gerontology and Geriatrics of the University of Perugia (UNIPG) in Italy and the Sociedad Vasca de Minusválidos of Bidaideak in Spain. Written informed consent was provided by the patients or their legal guardians. Ethical approval was obtained by the ethics committees of MUV, UNIPG and University of Basque Country. In each clinical center, 10 patients with a diagnosis of mild cognitive impairment (MCI) due to AD or mild AD according to the National Institute on Aging and Alzheimer’s Association (NIA AA) criteria [13] with a Mini-Mental-State-Examination (MMSE) between 24 and 28 (inclusive) were recruited. Furthermore, we defined a cutoff score equal or below 5 in activities of daily living according to the Lawton instrumental ADL score [14] as an inclusion criterion to ensure the ability of the participants to perform tasks important for the evaluation of MEMENTO. Subjects living with their spouse or in a family context, as well as subjects living alone with an informal supervisor (e.g., son, daughter or niece) were included in the trial. The caregivers were strongly involved in the field trials. In each study center, the ten participants were divided into a MEMENTO testing group (TG) and a control group (CG). Age, sex and MMSE were matched between the groups. The TG used the MEMENTO system while the CG used traditional means to organize their everyday life (e.g., calendars, post-its and notebooks). A peer contact person accompanied the users throughout the test period and was in close contact with the TG and the CG at each study site.

### Baseline data collection

Additional information was collected about each patient and respective caregiver. The cognitive reserve was determined using the Cognitive Reserve Index (CRI), a concept used to explain differences between the individuals in their capacity to cope with or compensate for pathology [15]. Technical proficiency of patient and caregiver, which refers to the skills required to operate an information system (i.e., a hardware/software solution), has been evaluated using a Likert scale based on participant information and usual use of technical devices at the beginning of the trials. In addition, information about the general attitude towards technology was collected, since it can be assumed that it is an important factor whether the patient likes or dislikes technology.

### MEMENTO prototype

**Fig. 1:**
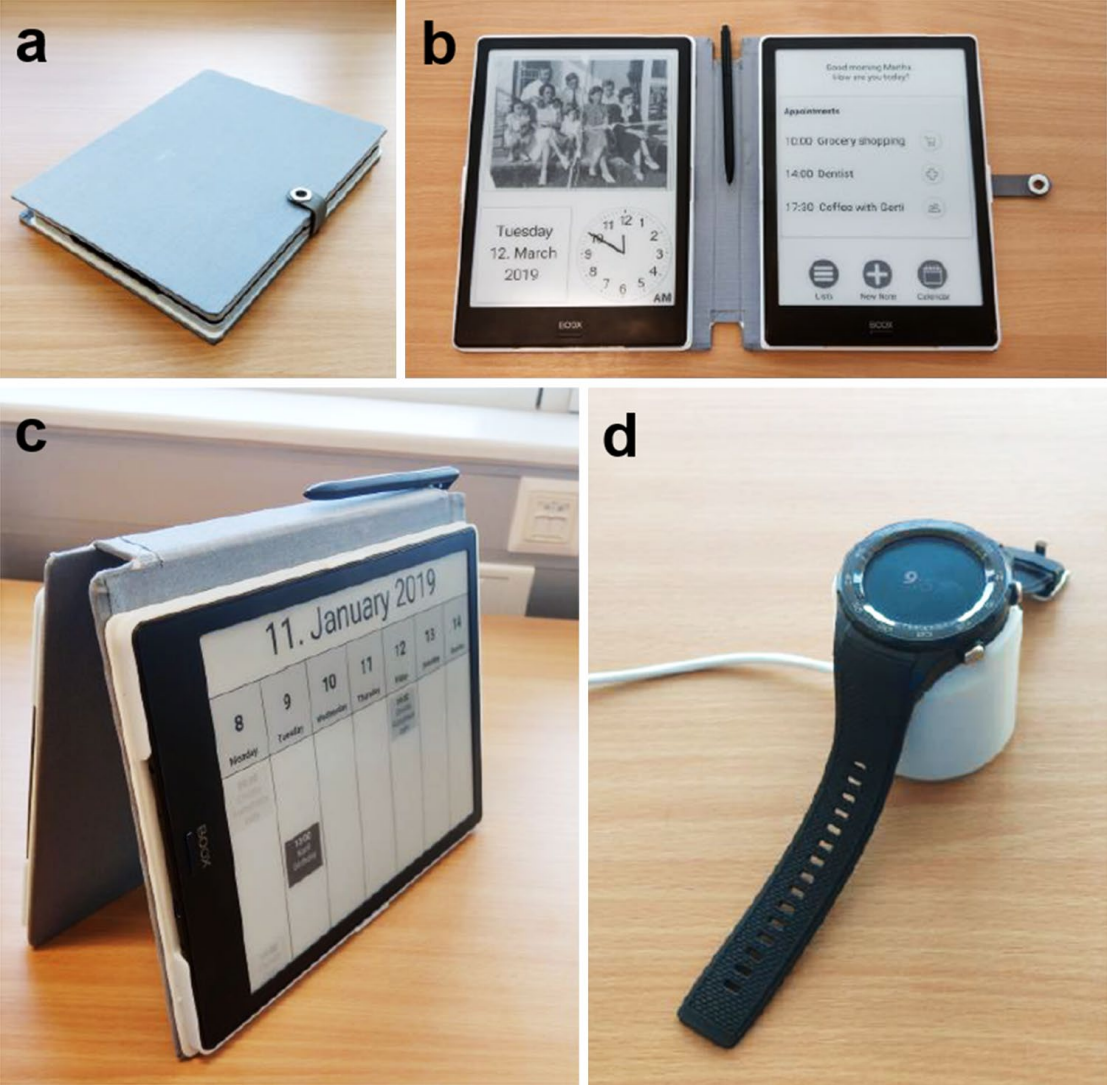
The MEMENTO prototype consists of a unit of two interlinked assistive devices. **a** The main device was modeled after an analog notebook to create familiarity and avoid stigmatization. **b** Consisting of two communicating e-ink tablets, it is used as a digital notebook, with great attention paid to the specific needs of dementia patients, such as large font and clear language and symbols, but also individualization by means of personal photographs. **c** When not in use, it can function as a calendar. The design modeled on familiar, analog desktop calendars. **d** The all-day device is a smartwatch that communicates with the main device via a cloud and can thus also be used independently. It reminds the user to take medication and keep appointments, can display and read out lists, make calls and inform relatives of the patient’s position via GPS when needed. The charger was designed to be stable for easy handling

A prototype of the MEMENTO system was used for the field trials, optimized based on previous feedback obtained from patients, caregivers and different stakeholders, e.g., physicians and dementia support groups. The system was carefully designed, taking traditionally used memory aids of dementia patients such as analogue calendars and notebooks into consideration to create a non-stigmatizing, archetypal object with high user acceptance [11, 12]. MEMENTO was designed as a unique hardware and software system comprised of two hardware components that act as one unit and a web interface. The main device is made up of 2 e-ink tablets with handwriting recognition housed in a bookcase that can either be carried similarly to a traditional diary or hung on a wall, thus resembling the tools patients are used to. The all-day device is a commercial smartwatch that assists the user both in- and outside of their home setting. It features a SIM card slot built-in and is able to place calls by itself. The prototype is shown in Fig. 1. Furthermore, a web interface provides caregivers the possibility to setup and monitor the system. Information on medication and appointments, as well as corresponding images (e.g., photography’s of the medication box and of important contact persons) and emergency contact details can be entered and managed via the caregiver interface.

The following features, based on six use cases from the requirement evaluation phase, were implemented for the field trials: (i) Medication: participants can enter their medication and set reminders, which both appear on the main and all-day device. Upcoming medication is shown on the home screen of the main device and can be looked up on the all-day device. (ii) Appointments: participants can enter appointments including an image and reminders, which appear both on the main and all-day device. Upcoming appointments are shown on the home screen of the main device and are synchronized with the all-day device. (iii) Getting ready: participants can create lists, e.g., packing lists for certain activities. The lists can again be viewed on both devices and can be ticked off. The all-day device can read the list aloud. (iv) Shopping: participants can create shopping lists. They are similarly designed and implemented as the getting ready lists. (v) Lost outside/lost at home: via a button on the all-day device, the current date, time, position and further activities of the day are read aloud by the smartwatch. The user is then asked if he/she wants to contact a caregiver. If needed, the caregiver is called automatically and additionally receives a text message containing the current position of the user. (vi) Panic: via a button on the all-day device, the caregiver is called automatically and again receives the current position of the user via text message.

### Study procedure

The study was presented to the TG and CG in separate workshops. Participants were introduced to the process of the field trials and methods used. Subsequently, each patient of the TG and (if available) his/her caregiver have been visited at home, where the system was installed and they were given the opportunity to try functionalities with guidance and support of their peer contact. Throughout the trials (12 weeks), the participants of the TG and CG were motivated bi-weekly in form of phone calls or meetings. The field trials were concluded by individual meetings with the participants to collect feedback and data and a group meeting (TG and CG separately) to share their experience with MEMENTO. A timetable of the field trial process is shown in the supplementary Table 1 (Online Resource).

### Quantitative and qualitative evaluations

Quantitative outcome measures concerned functional status, quality of life, caregiver burden, psychiatric symptoms, engagement and usability. Moreover, qualitative data were collected using interviews and diaries. Frequency of use of the system was also monitored using log files. A final focus group meeting was held to review the system and the trial phase.

#### Quantitative outcome measures

Quantitative outcome measures were pre-defined as the following: (i) World Health Organization Disability Assessment Schedule 2.0 (WHODAS 2.0) [16, 17], (ii) the Quality of Life—Alzheimer’s Disease scale (QOL-AD) [18], and (iii) the Alzheimer Disease Cooperative Study—Activities of Daily Living (ADCS-ADL) [19]. They were assessed in the TG and CG in both patients and primary caregivers, while the ADCS-ADL, concerning patient’s functional status, was administered only to the caregivers. Furthermore, we assessed (iv) Neuropsychiatric Inventory (NPI) [20], (v) Caregiver Burden Scale (CBI) [21], (vi) User Engagement Scale (UES) [22] and (vii) System Usability Scale (SUS) [23]. UES and SUS were assessed in patients and caregivers of the TG at the end of the trial, since the questions were directly related to the performance of the system. All other quantitative outcome measures were collected at the beginning and the end of the trials. The ADCS-ADL questionnaire was additionally performed in-between after 8 weeks (T0.5). Further information on quantitative outcome measures is provided in the Online Resource.

#### Qualitative outcome measures

Interviews, particularly indicated to evaluate usability in empirical studies involving dementia patients [24], were performed with all the participants and repeatedly conducted during the test phase. Procedures were standardized between clinical sites. In the beginning of the trials, data on strategies to remember were collected in an individual meeting in TG and CG. Diaries were distributed and both groups were asked to note down their strategies to remember during the trial period and in which situations they are used each day. Patients in the TG were additionally asked to record the use of the MEMENTO system. The effective use of the system was also monitored by means of the log file considering the daily frequency of use. During the trials, participants were called bi-weekly to motivate them and to collect feedback from diaries. The TG was visited at home in the middle of the trials (after 6 weeks). In the home visits, peer contacts evaluated potential user specific and technical problems with the MEMENTO device, went through the scenarios of the use cases and set tasks to test the usability with participants of the TG. Effectiveness, efficiency and satisfaction were considered as qualitative parameters [25]. The CG was called by their peer contacts and asked about their strategies in the same situations.

For the focus group meeting at the end of the trial, guiding questions were developed based on a consensus between the experts involved in the MEMENTO project and considering the Framework for Design Thinking for older people proposed by Wilkinson and Gandhi [26]. The objective was to sum up the users’ experience with the MEMENTO system at the end of the trials. Focus groups at clinical centers were performed by a moderator (E.S. in Vienna, M.P. in Italy and O.A. in Spain) and an observer in each group and were transcribed and reviewed for accuracy based on digital recordings. To be considered a prominent aspect, a theme had to be cited by two or more participants.

### Statistics

Statistics were performed using SPSS v17 and GraphPad Prism v8.0.1. Participant characteristics were evaluated using Mann–Whitney *U* tests. For outcome measure analysis, non-parametric tests were performed. The delta value (T1–T0; T1 representing data at the end of the trial, T0 representing data at the beginning of the trial) was calculated for quantitative outcome measures and Mann–Whitney *U* test was performed to compare the rate of change between TG and CG in patients and caregivers after 3 months. Correlations of age, sex, MMSE, technical proficiency, CRI, stimulation by caregiver and attitude towards technology with days of MEMENTO use was analyzed (Spearman’s rank correlation). Correction for multiple comparisons was performed using Bonferroni or Holm–Sidak method.

## Results

### Participant characteristics

Thirty patients participated in the study. In the TG, the median age was 72 years (IQR = 20) with a median MMSE of 27 (IQR = 2). The group consisted of nine female (60%) and six male (40%) participants. In the CG group, seven female (47%) and eight male (53%) patients participated, with a median age of 74 years (IQR = 8). The median MMSE was 27 (IQR = 2). There was no significant difference of age (*p *= 0.72) or MMSE (*p *= 0.80) between the groups. One participant in the Austrian TG terminated participation in the study at an early timepoint (4T_AT). Detailed information is provided in supplementary Table 2 (TG) and 3 (CG) of the Online Resource.

### Quantitative outcome measures

**Fig. 2:**
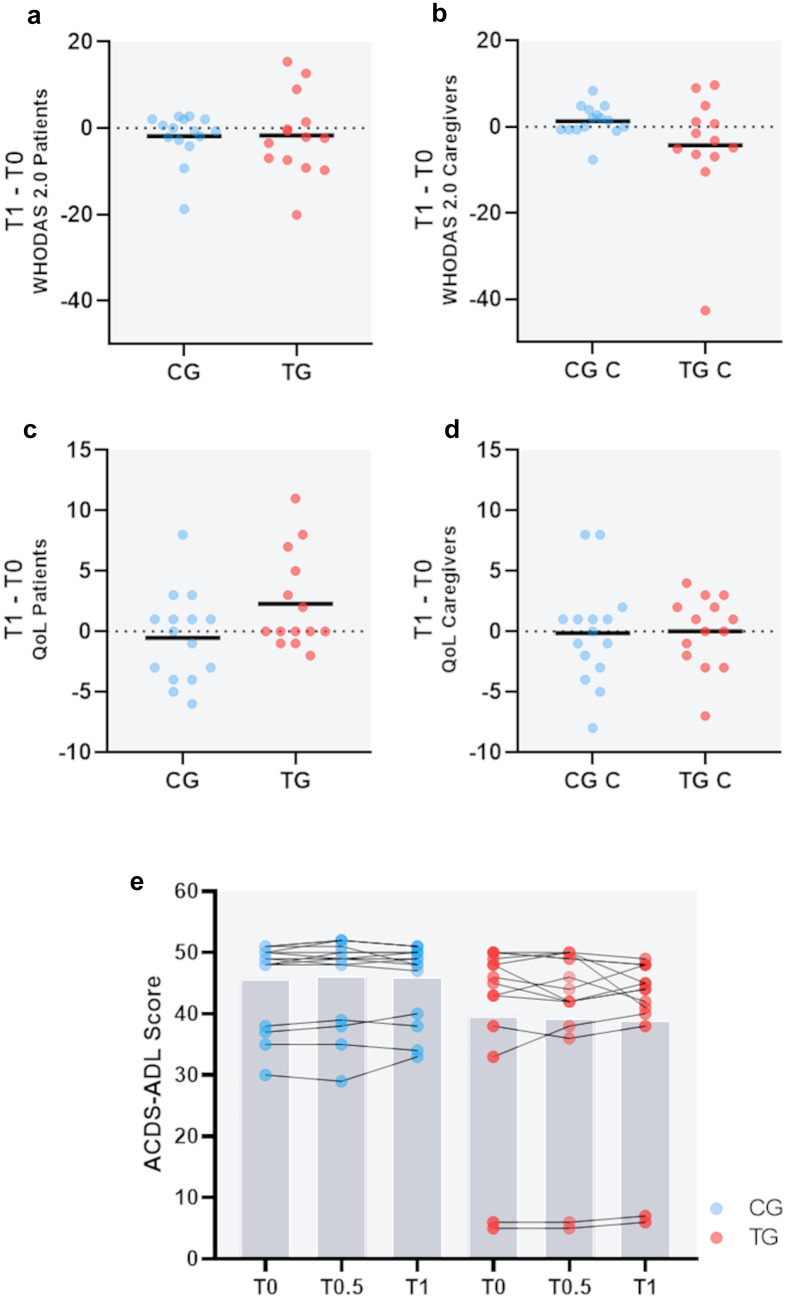
Evaluation of quantitative outcome measures. Delta values (T1–T0) show changes of the measures over time (12 weeks); dots represent single users, lines indicate means. **a** World Health Organization Disability Assessment Schedule 2.0 (WHODAS 2.0) of the patients (nCG = 15, nTG = 14; *p *= 0.5) and **b** of their caregivers (nCGC = 13, nTGC = 15; *p *= 0.1). Negative values indicate a decrease in disability. **c** Quality of life (QoL) of the patients (nCG = 15, nTG = 14; *p *= 0.1) and **d** of their caregivers (nCGC = 15, nTGC = 14; *p *= 0.6). Positive values indicate an increase in life quality. **e** Alzheimer Disease Cooperative Study-Activities of Daily Living (ACDS-ADL) score of the patients (nCG = 15, nTG = 14; *p *= 0.2). Higher values indicate more activities of daily living (range 0–53). Mann–Whitney *U* test was performed to compare delta values between the groups

In our first analysis, we surveyed the current condition of the patients and potential changes over the testing period by evaluating quantitative measures taken before and at the end of the field trials. In terms of disability, there was no significant difference in change of the WHODAS 2.0 over time when comparing the two groups (shown in Fig. 2a). Two patients in the TG showed high scores of disabilities further elevating over time, indicating a generally higher burden of disease in these patients. Likewise, there was no significant difference between the caregivers of the groups (shown in Fig. 2b). We also assessed the health-related quality of life in patients and their caregivers. High scores in the QOL-AD questionnaire indicate a better quality of life. There was no significant difference in change of life quality in the patients and caregivers over time (shown in Fig. 2c and d, respectively). A decline in the ability to perform activities of daily living is the everyday manifestation of cognitive and behavioral deficits. In the ADCS-ADL questionnaire, high scores indicate a higher grade of activity in daily living. There was no significant difference in change in the course of the field trials, as shown in Fig. 2e.

**Fig. 3:**
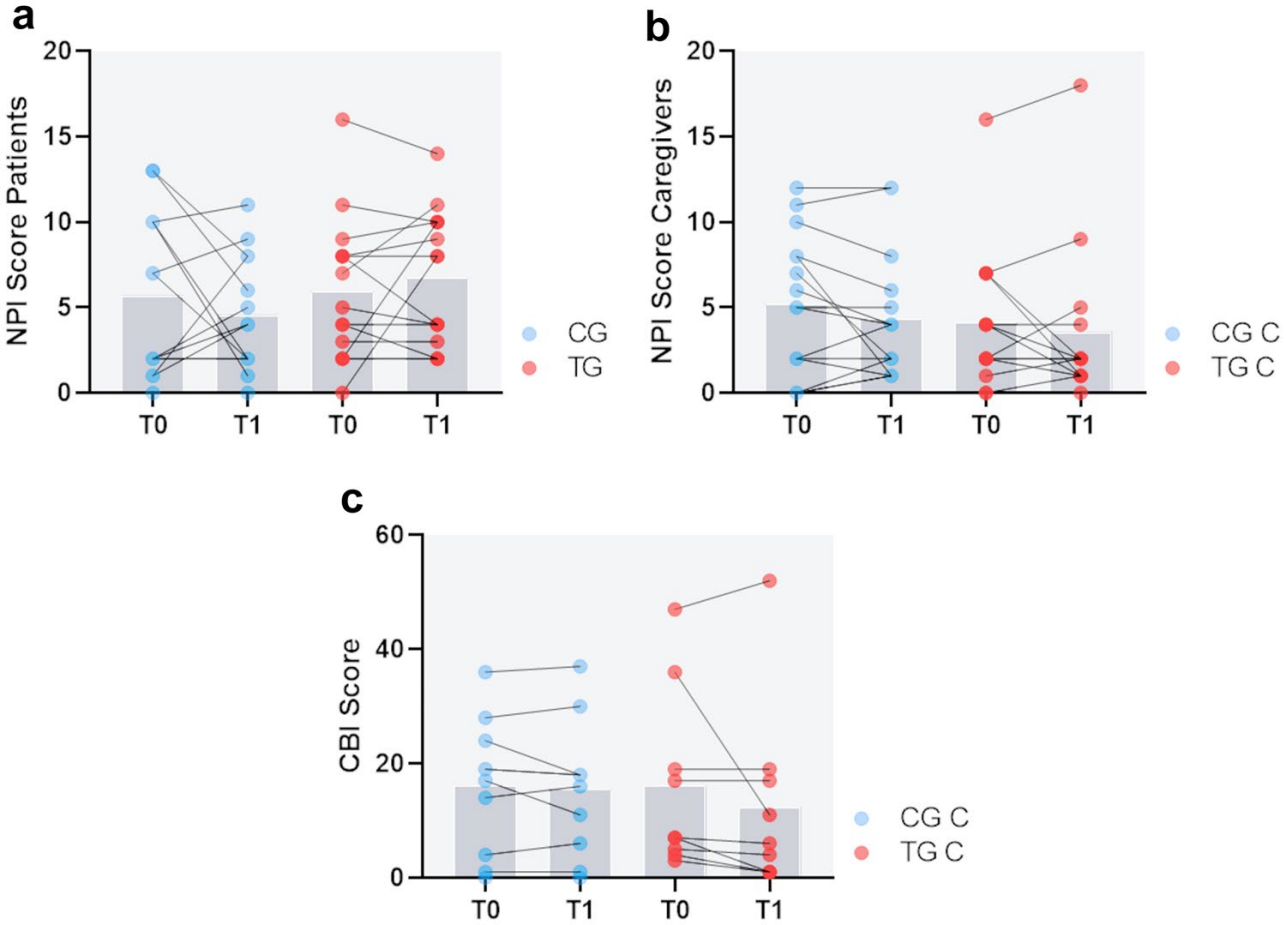
Evaluation of quantitative outcome measures. Neuropsychiatric inventory (NPI) and caregiver burden (CBI) analysis. The scatter plot shows scores at T0 and T1 (after 12 weeks, dots represent users), including the mean values as bars. **a** NPI of patients (nCG = 15, nTG = 14; *p *= 0.7) and **b** NPI distress of their caregivers (nCGC = 15, nTGC = 14; *p *= 0.2). Increasing values indicate an increase in neuropsychiatric burden in patients and increase in distress in caregivers. **c** CBI of the caregivers (nCGC = 10, nTGC = 9; 0.3). Increasing values indicate an increase in caregiver burden. Mann–Whitney *U* test was performed to compare delta values (T1–T0) between the groups

In addition, we aimed to evaluate neuropsychiatric disturbances in the patients before and after the trials, as well as the distress of their caregivers caused by such symptoms. High scores in the NPI questionnaire indicate a higher grade of neuropsychiatric symptoms in patients with dementia, as well as caregiver distress. There was no significant difference in change in the course of the field trials in both patients and caregivers of the TG and the CG. Furthermore, we analyzed the caregiver burden in general, where high burden is indicated by high scores in the CBI questionnaire. Again, there was no significant difference in change in the course of the field trials in caregivers of the TG and the CG. Figure 3 shows that there was a great variability in those measures across all groups over time.

An important outcome measure regarded the MEMENTO system itself in terms of usability and engagement of the participants. The UES questionnaire was filled in by patients and caregivers of the TG group (*n* = 11 and *n* = 9, respectively). Higher mean scores in the UES refer to higher engagement (scale = 1–5). Ratings on the core purpose of the MEMENTO system were mostly positive. The feeling of involvement (FI) was rated above average and the patients showed high curiosity and interest in the MEMENTO system (novelty, NO). Endurability (EN), esthetics (AE) and perceived usability (PU) were also rated above average. Both patients and caregivers assigned the lowest scores to focused attention (FA), and all the other aspects were rated above average by the caregivers with highest scores to novelty and esthetics, as shown in Table 1. The SUS was fully rated by 8 patients and 10 caregivers and showed a high variability. The low number of completed SUS scores might be due to difficulties of the patients to complete the questionnaire, as it was administered towards the end of the meeting and concentration was already waning. Several participants missed some answers or caregivers took over the answering of the questions. In either case, results could not be further considered for SUS analysis. The median SUS score of completed questionnaires was 47.5 (IQR = 16.87) and 50 (IQR = 13.12) out of 100 as rated by patients and caregivers, respectively.

**Table 1:** User engagement scale (UES)^a^

Subscales	Patients (*n* = 11) mean (SD)	Caregivers (*n* = 9) mean (SD)
Focused attention	2.3 (0.6)	2.8 (0.6)
Felt involvement	3.1 (0.5)	3.3 (0.5)
Novelty, curiosity and interest	3.5 (0.6)	3.9 (0.2)
Endurability	2.9 (0.5)	3.5 (0.3)
Esthetic appeal	3.4 (0.4)	3.7 (0.3)
Perceived usability	3.1 (0.7)	3.4 (0.1)

^a^UES ranges from 0 to 5, high scores indicate strong approval

### Evaluation of MEMENTO use

Participants of both TG and CG used several traditional memory aids in daily life, mainly calendars, post-its, agendas and pillboxes. Due to the specific age structure of our testing cohort, technical memory aids were seldom used. Therefore, participants expressed the need to gain more familiarity with MEMENTO before integrating the system as a routine in everyday life. Traditional strategies were experienced to be more familiar, intuitive, faster and secure at the moment, an aspect that limits the willingness to replace them with a new method regardless of its efficiency.

In general, the TG used the MEMENTO system with a weekly frequency. The information provided by the participants coincided well with the records monitored via log files, as shown in supplementary Table 4 (Online Resource). The most frequently used part of the MEMENTO system was the main device. As reported by the patients and caregivers, scheduling appointments was the most frequently used function, followed by shopping lists. The use case “getting ready” was rarely mentioned by the users. Some participants of the TG used the panic features as a fast means to contact their caregivers. Analysis of factors potentially influencing the frequency of use revealed a significant correlation with positive attitude towards technology (*r *= 0.723, *p *< 0.05), as shown in Table 2. Interestingly, there was no significant correlation with other variables, such as higher age or stimulation by the caregiver. To identify possible further correlations of variables and characteristics of our TG, a correlation matrix was created. Significant correlations of positive attitude towards technology with high technical proficiency (*r *= 0.789), higher age with MMSE (*r *= 0.614) and advanced age with technical proficiency (*r *= − 0.672) did not remain significant after correction for multiple testing. Notably, there was also no correlation between age and attitude towards technology (shown in suppl. Figure 1, Online Resource).

**Table 2:** Frequency of MEMENTO usage in correlation with patient characteristics

Variables	Spearman *r*	95% CI	*p* value	Adjusted *p* value^a^
Age	− 0.337	− 0.7437–0.2525	0.237	0.803
Sex	− 0.179	− 0.6583–0.4028	0.554	0.982
MMSE	0.062	− 0.4975–0.5855	0.831	0.988
Cognitive reserve	− 0.085	− 0.6003–0.4802	0.774	0.988
Technical proficiency patient	0.414	− 0.1671–0.7811	0.142	0.657
Technical proficiency caregiver	0.018	− 0.5299–0.5559	0.952	0.988
Stimulation by caregiver	− 0.152	− 0.6422–0.4260	0.615	0.982
Attitude towards technology	**0.723**	**0.2957**–**0.9090**	**0.006**	**0.047**

Significant correlation (*p* < 0.05) is highlighted in bold font

^a^*p* values were adjusted for multiple testing using Holm–Sidak correction;

### Qualitative feedback

In general, the overall design of MEMENTO was received well. Both devices were appreciated to look like archetypal objects and hence being not stigmatizing, which was a major concern.

Cognitive, physical, emotional and social support were attributed to the MEMENTO system by the participants. In contrast to traditional methods to remember, MEMENTO obligates people to write and categorize important aspects such as time, date and place, leading to secure disposition of important information and participants appreciated that MEMENTO acts as a reminder. Due to the “lost outside” and “panic” functionalities, users experienced a higher sense of security in daily life. Participants reported that cognitive impairment reduces social life. Feelings of shame, insecurity and sometimes fear of losing orientation can confine people at home. These aspects have negative impact on the health status, while maintaining or increasing social contacts promote wellbeing. MEMENTO’s support might help to promote the social life, also for the caregivers.

Participants furthermore attributed the system with a high value for the health status and quality of life and considered MEMENTO important as a support to maintain independence. People with mild dementia could live independently for an extended timespan and participants emphasized that the system could be very useful for persons living alone. Regarding caregivers, participants agree that their burden could be reduced by the monitoring possibility and the perception of a higher level of independence in the primary user.

Caregivers are considered very important to sustain the initial effort of training and to support the use of the system. Some users split up the system, with the caregiver adding data to the main device and the dementia patients mainly using the all-day device. Compared to writing on paper, writing on an e-ink surface was perceived as less convenient. The learning procedures required an initial effort, which not all participants were able to sustain so that caregiver’s motivation was necessary. Technical problems were the most cited factor that could limit the use of the system.

A general reflection on technologies showed that some participants considered technologies also as dangerous since memory strategies are delegated to a technological system. However, participants agreed that relying on technology guarantees greater independence and the benefits prevail the disadvantages. In the participants view, despite traditional methods are more familiar, in an increasingly digital society and for the next generation of older adults, MEMENTO and similar systems could be very important in our digital society and especially for the coming generation of older adults.

## Discussion

In the last decade, several scientific research and development studies addressing technologies for smart and healthy living in the elderly have been conducted. The present study explored the user experience of MEMENTO, an assistive technological system in daily life, during a 3-month field trial with persons diagnosed in early stages of cognitive decline. A mixed method strategy was used, including both quantitative and qualitative data.

The frequency of use of MEMENTO significantly relied on general attitude towards technology and not on age alone. This was highlighted by the correlation assessment of log files and user characteristics and the fact that higher age was not negatively related to days of use in our cohort, despite the test group (TG) included also oldest old participants, often underrepresented in research concerning new technologies [27]. This suggests that motivation and positivity towards technical devices are the most important factors to consider when developing a technical device for older adults. The importance of an individual’s attitude in active aging has been shown previously and learning, as well as using technical skills, depends particularly on a need for it seen by users [28, 29]. When introducing a new technical device, the user should, therefore, not only have a positive attitude towards the solution, but also be convinced of its prospective usefulness.

In general, participants attributed the MEMENTO system with cognitive, physical, emotional and social support. The overall experience was rated to be a success on average and users declared willingness to engage with the system in the future, especially in a further developed stage. One of the major concerns of users was stigmatization; therefore, both devices were appreciated to look like archetypal objects. Technical problems were mentioned to be a potential reason for abandoning the system and support of the caregivers in operating the system was important for the participants. Traditional strategies were experienced to be more familiar, intuitive, faster and secure at the moment, as also reported in previous studies concerning assistive technology in dementia patients [30].

A limitation of this study is the small number of participants and the short duration of the trial, making it difficult to observe clear trends in the quantitative outcome measures, considering that familiarity is a crucial factor for usability of a product [25]. Moreover, data on factors independent of MEMENTO that positively or negatively influence quality of life, activities of daily living or caregiver burden (e.g., relationship problems or interim changes in health) were not systematically collected. The heterogeneity of the sample (e.g., in regard to cognitive reserve, technical proficiency and attitude towards technology) might have additionally contributed to an absence of changes in quantitative outcome measures. However, our study represents an attempt to evaluate the devices in users’ home environments focusing on qualitative outcomes for everyday life rather than simply consider the effectiveness of the electronic memory aid, as it has been recently requested in assistive technology research [31].

The results of the field trials are comparable to previous studies on similar solutions [32], underlining the importance to involve people with dementia in the process of participatory design to realize devices that are accepted and relevant to their needs and to prevent failure of assistive technology implementation [8–10]. The recent SARS-CoV-2 pandemic highlights the increasing interest and importance of technological systems to promote and maintain independency on the one hand, and to have a safety net on the other [33]. However, such systems can only provide assistance if they are accepted by the users and well tailored to their needs. Although “focused attention” is not the main purpose of the system, this aspect will be interesting for further development, such as integrating games or including more personalized content to engage end-users. A stronger positive association with MEMENTO might lead to more interaction and thus a higher acceptance and commitment to the system. In addition, previous studies have shown that games can promote wellbeing of older persons and motivate them, e.g., by cognitive stimulation, physical activity and enjoyable experiences [34]. For a heterogeneous disease group like dementia, including various co-morbidities due to the typically advanced age, several options of adaptation to disabilities should exist, such as alternatives for the handwriting recognition feature. Notably, participants of the test group personalized the use of the system (e.g., the use of the “panic” feature to call the caregiver), demonstrating that dementia patients have their own strategies and ideas on how the problem should be solved, as also reported previously [30].

The thorough evaluation of field trials provided in this study will help the development of the MEMENTO prototype into a fully functional system that meets the needs of end-users. Most importantly, sharing our findings serves as a valuable resource for other projects aiming to develop assistive technologies for persons living with dementia.

## Supplementary Information

Below is the link to the electronic supplementary material.

Supplementary file1 (PDF 985 KB)

## Data Availability

Data are available on request by contacting the corresponding author (elisabeth.stoegmann@meduniwien.ac.at).

## Code availability

Data are available on request by contacting the corresponding author (elisabeth.stoegmann@meduniwien.ac.at).
