# Supplementary material for: User experience and acceptance of a device assisting persons with dementia in daily life: a multicenter field study
Source: Aging Clin Exp Res. 2021 Nov 11;34(4):869–79. doi: 10.1007/s40520-021-02013-8 (PMC8581127; doi:10.1007/s40520-021-02013-8)
Supplement: Supplementary file 1 — Supplementary file1 (PDF 985 KB) [file 40520_2021_2013_MOESM1_ESM.pdf]

Online Resources:

User experience and acceptance of a device assisting persons with dementia in daily life: a multicenter field study

Theresa König[1], Martina Pigliautile[2], Oscar Águila[3], Jon Arambarri[4], Christophoros Christophorou[5], Matteo Colombo[2], Argyris Constantinides[5], Rosario Curia[6], Kathrina Dankl[7], Sten Hanke[8], Christopher Clemens Mayer[9], Stefan Moritsch[10], Markus Müllner-Rieder[9], Fritz Pernkopf[11], Christian Schüler[12], Maria Stillo[6], Patrizia Mecocci[2], Elisabeth Stögmann[1*]

[1] Department of Neurology, Medical University of Vienna, Vienna, Austria
[2] Department of Medicine and Surgery, Section of Gerontology and Geriatrics, University of Perugia, Perugia, Italy
[3] Bidaideak - Sociedad Vasca de Minusválidos, Bilbao, Spain
[4] VirtualWare 2007 S.A., Basauri, Spain
[5] Citard Services Ltd., Nicosia, Cyprus
[6] Integris S.p.A., Innovation Lab, Rende and Pisa, Italy
[7] Design School Kolding, Kolding, Denmark
[8] University of Applied Sciences – FH Joanneum GmbH, Graz Austria
[9] Center for Health & Bioresources, Biomedical Systems, AIT Austrian Institute of Technology, Vienna, Austria
[10] bkm design working group, Vienna, Austria
[11] Fritz Pernkopf Industrial Design, Vienna, Austria
[12] Wetouch e.U., Vienna, Austria

***Corresponding Author:**
Elisabeth Stögmann
Department of Neurology
Medical University of Vienna
E-mail: elisabeth.stoegmann@meduniwien.ac.at

Supplementary Tables

Week	Where?	How?	Why?
Initial	Clinic	Group Meeting	We want to present the MEMENTO project and the time schedule for the upcoming months
1	Home	Visit	We will visit you at your home to deliver the system, show you how to use it and talk about open points
3	Home	Phone call	We will call you to see how you are handling the system and if there are any troubles
5	Home	Phone call	We will call you to see how you are handling the system and perform a quick interview on your activities of daily living
7	Home	Phone call	We will call you to see how you are handling the system and if there are any troubles
8	Home	Visit	We will visit you at your home to talk about the system and the diary and simulate different situations together
10	Home	Phone call	We will call you to see how you are handling the system and if there are any troubles
11	Clinic	Group Meeting	We will invite you to take part in a workshop to discuss the MEMENTO system and your experiences together with other users
Final	Clinic	Individual Meeting	We will invite you to an individual meeting at our clinic to collect your feedback

Supplementary Table 1. Timetable of the field trials presented to the MEMENTO users
The table shows the structure of the field trials, which was presented to the TG. In the CG, interviews concentrate on traditional strategies to remember.

Supplementary Table 2. Characteristics of the MEMENTO testing group

Patient ID	Age	Sex	MMSE	TP Patient	TP Caregiver	Cognitive Reserve Index	Status Caregiver
**1T_IT**	70	M	26	low	medium	medium	wife
**2T_IT**	81	F	28	medium	high	medium	nephew
**3T_IT**	81	F	28	low	high	medium-high	daughter
**4T_IT**	67	F	24	low	low	medium	husband
**5T_IT**	61	M	28	medium	medium	medium-high	wife
**1T_AT**	52	M	25	high	low	medium	wife
**2T_AT**	72	F	28	medium	medium	medium-high	husband
**3T_AT**	60	F	27	high	high	medium-high	husband
**4T_AT**	77	F	27	low	high	high	husband
**5T_AT**	54	M	25	high	high	medium	wife
**1T_ESP**	70	M	26	high	medium	high	wife
**2T_ESP**	76	F	27	medium	low	medium-high	son
**3T_ESP**	80	F	28	low	high	medium-low	nephew
**4T_ESP**	81	M	28	low	medium	medium	wife
**5T_ESP**	84	F	27	low	low	medium-high	son

Summary of participants in the TG. The patient ID consists of patient number and group (T for TG) followed by the country code (IT = Italy, AT = Austria, ESP = Spain). TP: technical proficiency

Supplementary Table 3. Characteristics of the control group

Patient ID	Age	Sex	MMSE	TP Patient	TP Caregiver	Cognitive Reserve Index	Status Caregiver
**1C_IT**	78	M	28	low	low	medium	wife
**2C_IT**	67	M	25	low	high	high	wife
**3C_IT**	80	M	28	low	low	medium-low	wife
**4C_IT**	70	F	27	medium	high	medium	daughter
**5C_IT**	62	M	28	low	high	medium-low	daughter
**1C_AT**	72	F	27	low	medium	medium	husband
**2C_AT**	67	F	28	medium	high	medium-high	daughter
**3C_AT**	74	M	26	high	medium	medium	wife
**4C_AT**	73	F	25	medium	medium	low	husband
**5C_AT**	70	M	26	high	high	high	wife
**1C_ESP**	78	F	27	medium	low	low	daughter
**2C_ESP**	75	F	25	high	medium	medium-low	daughter
**3C_ESP**	82	M	26	low	low	medium-low	wife
**4C_ESP**	80	M	28	low	medium	low	daughter
**5C_ESP**	78	F	27	low	low	low	son

Summary of participants in the CG. The patient ID consists of patient number and group (C for CG) followed by the country code (IT = Italy, AT = Austria, ESP = Spain). TP: technical proficiency

Supplementary Table 4. Evaluation of MEMENTO usage

ID	Days of use	Congruence with diary	Use over time	Stimulated by caregiver	Attitude towards technology
1T_IT	15	good	-	yes	negative
2T_IT	24	good	+	yes	positive
3T_IT	19	good	-	no	negative
4T_IT	15	good	-	yes	negative
5T_IT	27	medium	+	no	positive
1T_AT	61	good	=	yes	positive
2T_AT	23	good	=	yes	positive
3T_AT	6	good	-	yes	positive
4T_AT	drop out	n.a.	n.a.	yes	negative
5T_AT	21	medium	=	yes	positive
1T_SP	25	good	+	yes	positive
2T_SP	25	good	+	yes	positive
3T_SP	28	good	+	yes	positive
4T_SP	3	medium	-	yes	negative
5T_SP	2	medium	-	yes	negative

The table shows the frequency of use of Memento as evaluated by log files. The patient ID consists of patient number and group (T for TG) followed by the country code (IT = Italy, AT = Austria, ESP = Spain). Frequency of use is expressed as day of use based on the log file reports. In general, there is a good correspondence between the frequency of use referred by the users in the diary and the log reports. Column “Implemented in time”: + indicates an increase; – indicates a reduction; = indicates a steady use over time. Other variables potentially related to the frequency are stimulation by the caregiver and general attitude towards technology.

Supplementary Figures

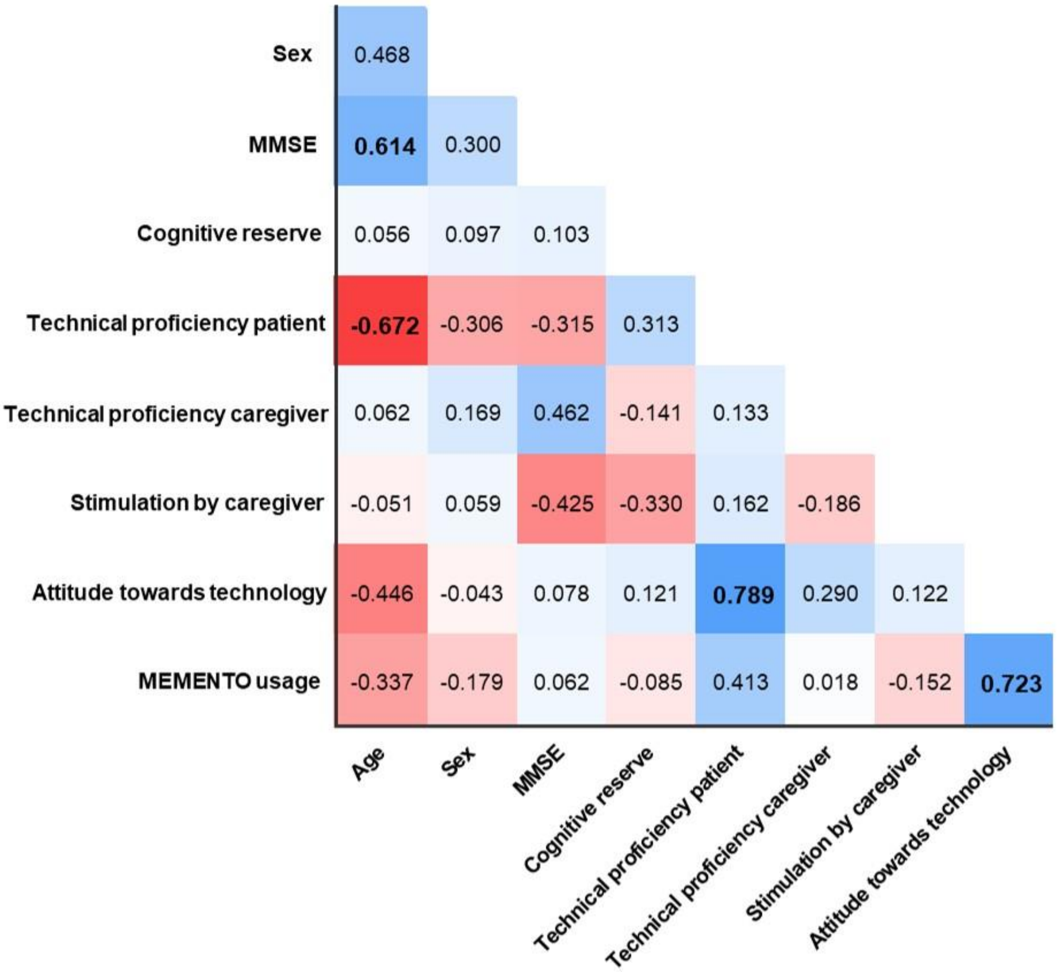

Supplementary Figure 1. Correlation matrix of user characteristics and MEMENTO use. Correlation heatmap of the TG group with r-values according to Spearman correlation (n=14). Significant correlations (indicated in bold) of positive attitude towards technology with MEMENTO usage (r=0.723) and high technical proficiency (r=0.789), higher age with MMSE (r=0.614) and advanced age with technical proficiency (r=-0.672) did not remain significant after correction for multiple testing, due to the quantity of variables.

Supplementary data

Quantitative outcome measures

The WHODAS 2.0 is a generic assessment instrument providing a standardized cross-cultural method for measuring activity limitations and participation restrictions, largely employed in geriatric settings (Bombin et al., 2012; Dernek et al., 2015). Specifically, the instrument is designed to evaluate the capabilities of the individual in six activity domains: (i) cognition (understanding and communication); (ii) mobility (ability to move and get around); (iii) self-care (ability to attend to personal hygiene, dressing and eating, and to live alone); (iv) getting along (ability to interact with other people); (v) life activities (ability to carry out responsibilities at home, work and school); (vi) participation in society (ability to engage in community, civil and recreational activities). For all six domains, the WHODAS 2.0 provides a profile and a summary measure of disability that is reliable and applicable across cultures in adult populations. To evaluate the WHODAS 2.0, the simple scoring option was adopted (Üstün et al., 2010). The Quality of Life – Alzheimer's Disease scale (QOL–AD) (Logsdon et al., 1999) was developed for individuals with dementia.

The Alzheimer Disease Cooperative Study-Activities of Daily Living (ADCS-ADL) (Galasko et al., 1997) is an inventory to assess activities of daily living for clinical trials in dementia.

The Neuropsychiatric Inventory (NPI) (Cummings et al., 1994) assesses neuropsychiatric disturbances common in dementia together with the amount of caregiver distress engendered by neuropsychiatric disorders (range of questionnaire 0-144).

The Caregiver Burden Scale (CBI) (Zarit et al., 1980) assesses perceived burden among caregivers of family members with dementia (range of questionnaire 0-88).

The User Engagement Scale (UES) (O'Brian and Toms, 2009) measures the user engagement by means of six dimensions of engagement. UES questions can be divided into the following categories: Focused attention (FA) is about the feeling to be absorbed in the interaction and losing track of time. Felt involvement (FI) refers to the sense of being "drawn in" and having fun, NO describes the novelty, curiosity and interest in the interactive tasks, EN the endurability, which means the overall success of the interaction and the users' willingness to recommend the system to others or engage with it in the future. The aesthetic appeal (AE) comprises questions about the attractiveness and visual appeal of the device and interface. Perceived usability (PU) refers to negative affect experienced and the degree of control and effort expended, such as "I felt frustrated while using MEMENTO". The questions were reverse coded in the analysis.

The System Usability Scale (SUS) (Brooke, 1996) is a quick measurement of how participants perceived the usability of a system. The mean score of the individual questions provided information about different aspects of usability.

References:

Bombin, I., Santiago-Ramajo, S., Garolera, M., Vega-Gonzalez, E.M., Cerulla, N., Caracuel, A., et al. (2012). Functional impairment as a defining feature of: amnestic MCI cognitive, emotional, and demographic correlates. *Int Psychogeriatr* 24(9), 1494-1504. doi: 10.1017/S1041610212000622.

Brooke, J. (1996). *SUS: a "quick and dirty" usability scale.* London: Taylor and Francis.

Cummings, J.L., Mega, M., Gray, K., and Rosenberg-Thompson, S. (1994). The Neuropsychiatric Inventory: Comprehensive assessment of psychopathology in dementia. *Neurology* 44(12), 2308–2314.

Dernek, B., Esmaeilzadeh, S., and Oral, A. (2015). The utility of the International Classification of Functioning, Disability and Health checklist for evaluating disability in a community-dwelling geriatric population sample. *Int J Rehabil Res* 38(2), 144-155. doi: 10.1097/MRR.0000000000000101.

Galasko, D., Bennett, D., Sano, M., Ernesto, C., Thomas, R., Grundman, M., et al. (1997). An inventory to assess activities of daily living for clinical trials in Alzheimer's disease. The Alzheimer's Disease Cooperative Study. *Alzheimer Disease and Associated Disorders* 11(Suppl 2)**,** S33–S39.

Logsdon, R.G., Gibbons, L.E., McCurry, S.M., and Teri, L. (1999). Quality of life in Alzheimer's Disease: Patient and caregiver reports. *Journal of Mental Health and Aging* 5(1)**,** 21–32.

O'Brian, H.L., and Toms, E.G. (2009). The development and evaluation of a survey to measure user engagement. *Journal of the American Society for Information Science and Technology* 61(1). doi: https://doi.org/10.1002/asi.21229.

Üstün, T.B., Kostanjsek, N., Chatterji, S., and Rehm, J. (2010). *Measuring Health and Disability: Manual for WHO Disability Assessment Schedule (WHODAS 2.0).* Geneva, CH: World Health Organization.

Zarit, S.H., Reever, K.E., and Bach-Peterson, J. (1980). Relatives of the impaired elderly: correlates of feelings of burden. *Gerontologist* 20(6)**,** 649-655. doi: 10.1093/geront/20.6.649.
